# Microbiota Orchestra in Parkinson’s Disease: The Nasal and Oral Maestros

**DOI:** 10.3390/biomedicines12112417

**Published:** 2024-10-22

**Authors:** Nádia Rei, Miguel Grunho, José João Mendes, Jorge Fonseca

**Affiliations:** 1 Egas Moniz Center for Interdisciplinary Research (CiiEM), Egas Moniz School of Health and Science, Campus Universitário, Quinta da Granja, 2829-511 Monte de Caparica, Portugal; mgrunho@egasmoniz.edu.pt (M.G.); jmendes@egasmoniz.edu.pt (J.J.M.); jfonseca@egasmoniz.edu.pt (J.F.); 2 Department of Neurology, Hospital Garcia de Orta EPE (HGO), 2805-267 Almada, Portugal; 3 Department of Gastroenterology, Hospital Garcia de Orta EPE (HGO), 2805-267 Almada, Portugal

**Keywords:** Parkinson’s disease, nasal microbiota, oral microbiota, microbiome, dysbiosis

## Abstract

Parkinson’s disease (PD) is characterized by the progressive degeneration of dopaminergic neurons, leading to a range of motor and non-motor symptoms. Background/Objectives: Over the past decade, studies have identified a potential link between the microbiome and PD pathophysiology. The literature suggests that specific bacterial communities from the gut, oral, and nasal microbiota may be involved in neuroinflammatory processes, which are hallmarks of PD. This review aims to comprehensively analyze the current research on the composition, diversity, and dysbiosis characteristics of the nasal and oral microbiota in PD. Methods: Through a comprehensive search across scientific databases, we identify twenty original studies investigating the nasal and oral microbiota in PD. Results: Most of these studies demonstrate the substantial roles of bacterial communities in neuroinflammatory pathways associated with PD progression. They also underscore the influences of microbiota-derived factors on key aspects of PD pathology, including alpha-synuclein aggregation and immune dysregulation. Conclusions: Finally, we discuss the potential diagnostic and therapeutic implications of modulating the nasal and oral microbiota in PD management. This analysis seeks to identify potential avenues for future research in order to clarify the complex relationships between these microorganisms and PD.

## 1. Introduction

Parkinson’s disease (PD) stands as the most prevalent neurodegenerative movement disorder and ranks as the second most frequent neurodegenerative disease [1]. Its incidence is estimated at 10–19 cases per 100,000 person-years [2], affecting approximately 1% of individuals aged sixty or older [3]. Although Parkinson’s disease (PD) is typically characterized as a neurodegenerative disorder primarily associated with aging, there is a subset of cases diagnosed between the ages of 21 and 40; these cases are commonly referred to as young-onset PD patients [4]. PD is a complex disease with multiple contributing factors that has emerged as one of the leading causes of disability worldwide, with the fastest-growing rates of prevalence, disability, and mortality among various neurological disorders [5,6]. With the increasing longevity of the population, the incidence of PD is expected to continue to rise in the coming decades [7].

Most PD cases (approximately 85%) are sporadic, with a hypothesized involvement of environmental factors alongside genetic and non-genetic risk factors. In familial cases, a genetic factor is evident, with specific genes having been identified as associated with hereditary forms of the disease. Among the most frequently mutated genes linked to familial PD are Leucine-rich repeat kinase 2 (LRRK2) and Parkin RBR E3 Ubiquitin Protein Ligase (PRKN) [8].

The two most well-known pathological hallmarks of PD are the deposition and aggregation of misfolded alpha-synuclein (aSyn) in the form of Lewy bodies and Lewy neurites and the loss of dopaminergic neurons in the substantia nigra pars compacta (SNpc) [9,10,11]. This leads to the development of several motor symptoms, such as bradykinesia, rigidity, resting tremor, postural unsteadiness, and gait abnormalities [12]. The diagnosis typically relies on the presence of these motor symptoms, which poses a significant clinical challenge, as they often manifest only when a substantial proportion (60–80%) of dopamine-producing neurons in the substantia nigra have already been lost [13].

PD involves several other non-motor symptoms, namely, depression, psychosis, cognitive impairment, sleep disturbance, and gastrointestinal dysfunction, the latter occurring in more than 80% of patients [14].

In recent years, several studies have shown an association between the dysbiosis of oral and nasal microbiomes and PD [15,16,17,18,19]. Moreover, individuals with PD present poorer oral health compared to the general population [20,21]. The oral cavity harbors a diverse community of microorganisms that is essential for maintaining oral health and systemic homeostasis. Alterations in the composition and function of the oral microbiota are linked to various systemic diseases, including PD [22], and oral dysbiosis may influence the pathogenesis of PD through the mouth–gut connection [22,23]. Studying the role of the oral microbiota in the development of PD may offer novel insights into the disease mechanisms and provide potential therapeutic targets.

The nasal microbiota is also attracting attention for its potential involvement in the development and progression of PD. Olfactory impairment is frequent in PD’s earlier stages, namely, in the prodromal phase of the disease. It is conceivable that the microbial community in the nasal passages may contribute to the appearance and aggregation of the misfolded aSyn protein, which is highly abundant in olfactory sensory neurons (OSN) [24]. Considering that olfaction is lost in approximately 90% of cases of pre-motor PD [25], the nasal cavity, along with the gut, could be one of the sites where PD neuroinflammation is triggered. This hypothesis, known as the “dual-hit hypothesis,” suggests that the nasal cavity serves as a pathway for pathogen invasion or toxin exposure [24,26,27]. However, the relationship between the dysbiosis of the oral and nasal microbiota and the pathogenesis of PD has been the subject of significantly less research than the relationship between gut dysbiosis and PD.

Given the scarcity of literature on this topic, we have opted for a narrative review approach. The aim of this review is to provide a comprehensive summary of the existing literature on the differences in the nasal and oral microbiota in relation to PD. In most studies, researchers have looked at the overall measures of biodiversity by analyzing species richness and distribution within individual samples (alpha diversity), as well as differences between groups (beta diversity) [28]. The therapeutic and diagnostic implications of these differences are also discussed to fill knowledge gaps and provide the basis for potential innovative therapeutic strategies.

## 2. Summary of Included Studies

**Figure 1:**
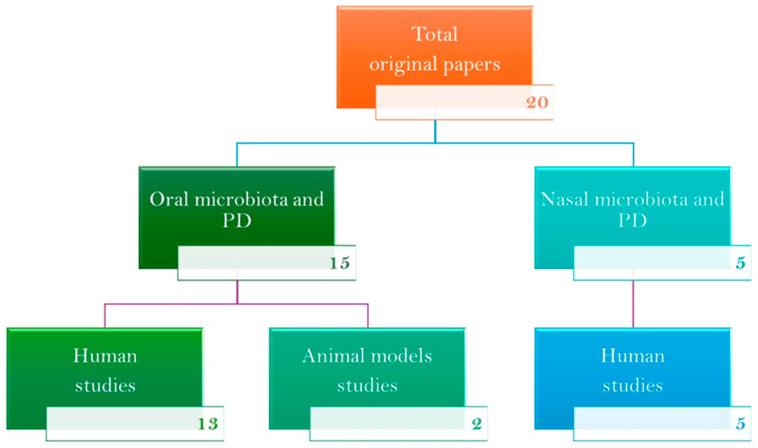
A flowchart outlining the 20 original studies that explored the association between nasal and oral microbiomes and PD. The results were categorized into two groups: studies examining nasal microbiota in humans (no literature was found for studies in animal models) and studies examining oral microbiota, which included research conducted in both human and animal models.

This review analyzes the original research articles that explore the compositions of the nasal and oral microbiota in subjects with PD. A comprehensive search was conducted in July 2024 across several databases, including PubMed, Embase, Google Scholar, Web of Science, and Scopus, for studies involving both human subjects and animal models. The search terms employed were “Parkinson’s disease AND nasal AND (bacteria OR microbiome OR microbiota)” and “Parkinson’s disease AND oral AND (bacteria OR microbiome OR microbiota)”. Only full-text articles published in English up to 31 July 2024 were included. The existing literature was compiled according to the selection criteria. The results were divided into two categories: studies of nasal microbiota in humans (no studies conducted in animal models were found) and studies of oral microbiota, including both human and animal model research. A total of twenty original papers were identified. Five studies investigated the nasal microbiota and PD in humans, thirteen studies explored the oral microbiota and PD in humans, and two studies focused on the oral microbiota and PD in animal models (Figure 1).

## 3. Nasal Microbiota in Parkinson’s Disease

### Human Studies

Our search of the literature identified five studies that explored the nasal microbiota in human persons with PD (Table 1). No studies addressing this topic in animal models were found.

Several studies have compared the nasal microbiota profiles of individuals with PD and healthy control individuals. While some studies have found an increased abundance of pro-inflammatory bacteria and a decrement of beneficial bacterial taxa in PD patients, others have found no significant differences in the compositions of the nasal microbiota between PD patients and controls.

A 2017 study [29] revealed that the nasal microbiota varies significantly between individuals, with most operational taxonomic units (OTU) being sparsely detected. Sex has been found to be the most influential factor shaping the structure of the nasal microbial community. After controlling for sex, only a few bacterial families have been identified as differentially abundant in PD patients, mainly comprising rare taxa. Interestingly, the abundance of a particular family, *Bacillaceae*, exhibits notable variations among patients undergoing levodopa therapy. The results indicate that the medications for PD, particularly levodopa, may exert a more pronounced influence on the bacterial families than the disease itself. 

In the same year, another study [15] found no significant differences in alpha or beta diversity between PD patients and control subjects. However, PD patients have shown a lower abundance of the genera *Marmoricola* and the family *Flavobacteriaceae*. *Marmoricola* is commonly found in environmental sources, while *Flavobacteriaceae* includes potential human pathogens. The authors acknowledge the limitations of the employed sampling methodology, particularly the inability to access the olfactory epithelium, which may have masked PD-related alterations [15].

A 2021 study has revealed the presence of dysbiotic and potentially proinflammatory microbiota in the deep nasal sinus cavities of subjects with PD differing from the controls [30]. The study shows a positive correlation between proinflammatory bacteria, including *M. catarrhalis*, and PD clinical features. The authors have suggested that future studies should try to establish a causal link between nasal microbiota dysbiosis and PD pathogenesis [30].

In the same year, a Li et al. study [31] showed that, compared with PD patients without constipation, constipated PD patients are at a higher risk of developing hyposmia and have a reduced response to subthalamic nucleus deep brain stimulation (STN-DBS) in terms of improving olfactory function. This occurrence may be significantly related to dysbiosis, as evidenced by a 16S examination of samples from the nasal mucosa of PD patients. PD patients with hyposmia, especially those with constipation, have lower bacterial abundance in the nasal mucosa and reduced diversity compared to the controls. The comparison between the hyposmia with and without constipation subgroups shows higher percentages of certain bacteria (*Stenotrophomonas*, *Lactobacillus*, *Neisseria*, and *Veillonella*) in the hyposmia/constipation group. This is consistent with previous findings in the oral microbiota of PD patients [32].

Contrarily, a 2022 study [33] found no significant differences in the alpha or beta diversity of nasal microbiota between PD patients and healthy controls, suggesting that the use of the nasal microbiota as a PD biomarker could be of limited value.

**Table 1:** This table summarizes the key studies investigating the nasal microbiota in patients with PD. The information includes the authors, year of publication, type of population studied, and main findings of each study.

Authors	Year	Population	Key Findings/Main Conclusions
Pereira et al. [15]	2017	PD patients vs. Healthy controls	No significant differences in alpha or beta diversity between PD patients and control subjects. PD patients showed lower abundance of the genera *Marmoricola* and the family *Flavobacteriaceae*.
Heintz-Buschart et al. [29]	2017	PD patients vs. idiopathic rapid eye movement sleep behavior disorder patients vs. Healthy controls	Nasal microbiota varies significantly between individuals. Sex is the most influential factor. Few bacterial families differentially abundant in PD patients, mainly rare taxa. *Bacillaceae* abundance varies with levodopa.
Pal et al. [30]	2021	PD patients vs. Healthy controls	There is a presence of dysbiotic and potentially proinflammatory microbiota in the deep nasal sinus cavities of PD subjects differing from the controls. There is a positive correlation between proinflammatory bacteria (e.g., *M. catarrhalis*) and the clinical features of PD.
Li et al. [31]	2021	PD patients with or without constipation vs. Healthy controls	PD patients with constipation have a higher risk of hyposmia and reduced response to STN-DBS for olfactory function. PD patients with hyposmia and constipation had lower bacterial abundance and reduced flora diversity in nasal mucosa compared to controls. Higher percentages of *Stenotrophomonas*, *Lactobacillus*, *Neisseria*, and *Veillonella* are found in the hyposmia with constipation group compared to the hyposmia without constipation group.
Li et al. [33]	2022	PD patients vs. Healthy controls	No significant differences in alpha or beta diversity of nasal microbiota between PD patients and healthy controls are found.

## 4. Oral Microbiota in Parkinson’s Diseases

### 4.1. Human Studies

Our search identified fifteen studies investigating the oral microbiota in PD, thirteen of which involved human subjects (Table 2). These various studies showed significant differences in the composition and diversity of the oral microbiota between patients with PD and healthy individuals.

A study conducted in 1994 compared three groups of 14 individuals: a control group, a group of PD patients who craved sweets, and a group of PD patients who did not crave sweets. The microbiota of plaque samples from the mandibular left second molar were analyzed. The study found no significant correlation between oral microbiota and clinical assessments. The isolation of *Actinomyces*, *Streptococcus mutans*, and *Veillonella* was more frequent than that of *Lactobacillus*, *Bacteroides*, and *Fusobacteria*. The percentage of *Streptococcus mutans* was significantly higher in both PD groups compared to the control group. No significant differences were observed in the frequencies of *Lactobacillus*, *Bacteroides*, *Fusobacteria*, *Veillonella*, and *Actinomyces* among the groups [34].

A 2003 preliminary study evaluated the presence of oral Gram-negative bacteria in 50 patients with PD. The researchers discovered that 16 patients exhibited the presence of six distinct Gram-negative bacteria, including *Escherichia coli*, *Klebsiella* spp., *Kluyvera* spp., *Serratia* spp., *Proteus* spp., and *Enterobacter* spp. The findings indicated that patients with oromuscular dysfunction demonstrated a higher prevalence of these bacteria compared to those with normal swallowing skills. No correlation was identified between disease severity and the presence of these bacteria [35].

A 2017 study [15] identified differences in beta diversity and in the abundance of specific bacterial taxa. A comparison between control and PD groups revealed significant differences in the abundance of various taxa, including potential oral pathogens, with some increasing (*Prevotella*, *Prevotellaceae*, *Veillonella*, *Solobacterium*, *Veillonellaceae*, *Lactobacillaceae*, and *Coriobacteriaceae*) and others decreasing (*Capnocytophaga*, *Rothia*, *Kingella*, *Leptotrichia*, *Actinomyces*, and *Leptotrichiaceae*) in PD patients. Additional taxa, including *Haemophilus*, *Neisseria*, *Gemella*, *Corynebacterium*, *Granulicatella*, and others, were found to be decreased in PD patients, whereas *Moryella* and *Erysipelotrichaceae* showed increased abundance. Irrespective of their periodontal disease status, males showed higher levels of potential oral pathogens than females. The abundance of *Porphyromonas* Otu000029 and an unclassified *Prevotellaceae* OTU was positively correlated with age, while the abundance of *Flavobacteriaceae* was negatively correlated, suggesting potential age-related changes that could be relevant to understanding both aging and PD.

A 2019 study [16] aimed to investigate changes in the oral microbiome during the early stages of PD using shotgun metatranscriptomic profiling involving 48 PD patients and 36 healthy matched controls. Saliva samples were collected and analyzed using next-generation sequencing to profile microbial RNA and host mRNA. No differences were detected in the overall alpha and beta diversities across subject groups. Nonetheless, regarding microbial genus and species differences, a total of 50 taxa exhibited significant abundance differences in PD subjects compared to controls, including bacteria, phages, and yeasts. These taxa served as effective classifiers in distinguishing PD subjects from controls. Most observed changes involved an increase in abundance, particularly among genera such as *Lactobacillus* and *Bifidobacterium*, as well as yeast species such as *Candida albicans*, *Candida dubliniensis*, and *Saccharomyces cerevisiae* in PD subjects. In contrast, only a few taxa exhibited decreases in abundance, such as *Buchnera*, *Candidatus*, and *Campylobacter*. The analysis revealed metabolic shifts in amino acid and energy pathways among oral microbiomes when comparing PD patients with controls. Moreover, the analysis of human salivary mRNAs revealed significant alterations in nine host mRNAs, with some being linked to brain function and correlated with changes in microbial taxa. Strong associations were observed between microbiota and functional measures, such as cognition, balance, and disease duration. These findings highlighted the potential of the oral microbiome as a readily accessible and informative microenvironment that could provide new insights into the pathophysiology of early-stage PD.

A 2021 study [17] analyzed the microbiota of PD patients and controls, focusing on samples from saliva and subgingival plaque. PD patients had lower alpha diversity in plaque samples, with a similar trend that was consistently observed in saliva samples. Patients with PD had no significant dental or periodontal health problems but presented a significant increase in the pro-inflammatory cytokine IL-1β in their gingival crevicular fluid. They also presented elevated levels of IL-1 receptor antagonist (IL-1RA) and a trend toward elevated levels of TNF-α, which was also observed in gingival crevicular fluid. Salivary microbial profiles were associated with cognitive status, salivary flow rate, and increased risk for periodontal disease. No significant differences in dental and periodontal parameters were observed between PD patients and controls. However, differences were observed in both salivary and subgingival plaque microbiota between PD patients and controls. The study found a correlation between the compositions of dental plaque microbiota and the severity of motor symptoms in PD patients in the early and mid-stages of the disease: PD patients presented a higher abundance of certain bacterial species, including *Streptococcus mutans*, *Kingella oralis*, *Actinomyces* AFQCs, *Veillonella* AFUJs, *Scardovia*, *Lactobacillaceae*, *Negativicutes*, and *Firmicutes.* Other species, such as *Treponema* KE332528 s, *Lachnospiraceae* AM420052s, and phylum SR1, were less abundant. These findings suggested that changes occurred in the oral microbiome during the early and middle stages of PD, even when maintaining good dental and periodontal health. The presence of local inflammation in the oral cavities of PD patients highlighted the need for further research into the relationship between oral dysbiosis, inflammation, and the pathophysiology of this disease.

A 2021 case-control study [36] utilized 16S rRNA gene sequences to compare the oral microbiota of PD patients with various oral-related symptoms to that of healthy controls. There were no significant differences in community richness between PD patients and controls based on alpha diversity measures, but beta diversity showed significant differences between hard and soft tissues in both groups. PD samples exhibited increased abundance of *Lactobacillus*, *Tannerella forsythia*, and *Prevotella intermedia*, as well as potential pathogenic oral species, including *Streptococcus pneumoniae*, *Mycoplasma orale*, and *Streptococcus constellatus*. Dysphagia, drooling, and salivary pH were identified as significant factors influencing soft tissue beta diversity and microbiota composition. *Streptococcus pneumoniae* levels were elevated in PD patients with dysphagia compared to the controls, and PD severity and medication status also impacted oral microbiota composition. This study highlighted the increased vulnerability of PD patients to aspiration pneumonia, which was often attributed to alterations in their oral microbiota. The authors suggested that the oral microbiota in PD maintained an overall microbial diversity and community richness, with noticeable differences between hard and soft tissue sites.

A 2022 study [33] found significant differences in microbiota composition between PD patients and healthy controls. These differences were particularly evident in the oral cavity and gut but not in the nasal cavity, even after adjusting for demographic factors such as age, sex, and body mass index. Alpha diversity indexes within the oral and nasal cavities did not show any significant differences. Beta diversity analysis showed significant differences in microbiota structure within the gut and oral cavity of PD patients compared to controls. PD patients exhibited higher levels of *Firmicutes*, *Bacilli*, *Lactobacillales*, *Streptococcaceae*, *Streptococcus*, *Streptococcus mitis*, unidentified *Prevotellaceae*, and *Prevotellaceae* in their oral microbiota compared to healthy controls. PD patients exhibited increased levels of *Firmicutes* not only in the oral cavity but also in the gut. This observation aligned with prior studies on the subgingival microbiome in periodontal disease, indicating a potential migration of *Firmicutes* from the oral cavity to the intestine [33]. In contrast, the control group showed a higher relative abundance of *Neisseria*, *Neisseria subflava*, unidentified *Gammaproteobacteria*, *Neisseiaceae*, *Gammaproteobacteria*, and *Proteobacteria* compared to the PD group. Several genera in the oral cavity exhibited significant positive correlations with clinical features, including scores on the Hamilton Anxiety Scale (HAM-A) and Hamilton Depression Scale (HAM-D). A correlation analysis suggested a connection between *Firmicutes* in the oral cavity and clinical symptoms like anxiety.

Another 2022 study [22] that aimed to investigate the interplay between the oral and gut microbiomes in patients with PD showed that the taxonomic composition of the oral microbiome in PD patients differed from that of the healthy controls. PD patients had a greater abundance of oral *Lactobacillus* compared to healthy controls. Oral *Lactobacillus* had the highest Linear Discriminant Analysis (LDA) score in PD patients, regardless of confounding factors such as disease duration and dysphagia. Previous studies [15,16,17,31] also consistently demonstrated an increase in oral *Lactobacillus* levels in patients with PD. However, the specific influence of oral *Lactobacillus* on the pathogenesis of PD remained unclear. The increase in oral *Lactobacillus* was observed to be associated with increases in intestinal pathogens, such as *Citrobacter*, *Klebsiella*, and *Enterobacter*. This suggested that oral *Lactobacillus* had the potential to travel to the gut and change the intestinal environment, promoting the colonization of pathogens.

In the same year, Zapala et al. [37] conducted a study to compare the oral microbiota in PD patients and healthy controls using 16S rRNA gene sequencing. The study revealed significant differences in the compositions of the oral microbiota. PD patients presented reduced levels of *Proteobacteria*, *Pastescibacteria*, and *Tenercutes*, while the genera *Prevotella*, *Streptococcus*, and *Lactobacillus* were found to be higher. PD patients also presented distinct dietary preferences from healthy controls, consuming more margarine, fish, red meat, cereal products, avocado, and olives. This research uncovered significant associations between certain foods and oral bacteria. Specifically, *Veillonella rogosae* showed a strong positive correlation with frequent consumption of leafy greens, while *Prevotella pallens* had the highest negative correlation with alcohol consumption.

A 2023 study [38] shed light on how PD impacted the subgingival microbiome. The study found a significant increase in bleeding upon probing PD patients with periodontitis compared to those with PD alone. Checkerboard analysis identified specific microbial taxa associated with PD and periodontal health. The authors showed that individuals with periodontal disease had higher levels of several types of bacteria, including *Treponema socranskii*, *Peptostreptococcaceae* [G-6] [*Eubacterium*] *nodatum*, *Parvimona micra*, *Prevotella melaninogenica*, *Lachnoanaerobaculum saburreum*, and *Streptococcus anginosus*. The PD patient group with periodontitis exhibited higher levels of *Streptococcus intermedia*, *P. nodatum*, *P. micra*, *Treponema denticola*, *L. saburreum*, *P. melaninogenica*, and *Campylobacter rectus* compared to the PD group in their deep pockets, while the level of *Aggregatibacter actinomycetemcomitans* was lower. In the PD patient group with periodontitis, *Schaalia odontolytica* and *A. actinomycetemcomitans* decreased, while *C. rectus*, *P. micra*, *Streptococcus constellatus*, *T. denticola*, *P. melaninogenica*, and *T. socranskii* increased in shallow pockets. Next Generation Sequencing (NGS) results showed minimal differences in alpha and beta diversity between the PD and PD patients with periodontitis groups. The authors suggested that periodontal disease affected the subgingival microbiome associated with PD, pointing to a possible link between PD and periodontal health.

Another 2023 work [39] investigated the changes in the saliva compositions in PD patients at different stages of cognitive impairment (CI), including mild cognitive impairment (PD-MCI) and full dementia (PDD), compared to healthy controls. By using 16S rRNA gene amplicon sequencing and metaproteomic profiling, the authors identified unique signatures in salivary compositions corresponding to different stages of CI in PD. Their innovative integration of metaproteogenomics revealed significant shifts in the salivary microbiome, protein translation machinery, and defense mechanisms, and it elucidated changes in the human proteome. The study found no significant differences in alpha diversity. However, beta diversity comparisons of saliva samples revealed notable distinctions across different stages of CI. A significant decrease in the *Neisseria* genus was observed as CI advanced, a trend also seen in PD patients, indicating a continuous decline in *Neisseria* with CI progression in PD. These findings emphasized the link between CI progression in PD and changes in both salivary microbial communities and protein expression profiles.

A 2024 study [40] aimed to investigate whether PD affected the oral microbiome associated with periodontitis. The study included three groups of participants: individuals with periodontitis and PD (P + PD), individuals with periodontitis without PD (P), and individuals who were healthy controls (HC) without any systemic health issues or periodontitis. The P + PD group had mild-to-moderate motor dysfunction, but plaque scores were similar to those without PD, indicating effective oral hygiene in the P + PD group. Significant differences in beta diversity were observed in saliva samples, with distinct patterns evident across different groups. Individuals diagnosed with periodontitis exhibited increased levels of *Mycoplasma faucium*, *Tannerella forsythia*, *Parvimonas micra*, and *Saccharibacteria* (TM7). In contrast, the P + PD group demonstrated higher abundance levels of *Prevotella pallens*, *Prevotella melaninogenica*, and *Neisseria* multispecies. Gender differences were observed, with a higher proportion of males in the P + PD group, which was consistent with the known prevalence of PD. In addition, the P + PD group had a higher mean age, which was consistent with the age-related increase in the prevalence of PD. The study highlighted significant changes in the oral microbiota associated with periodontitis in individuals with PD and underscored the importance of considering demographic and clinical factors in understanding the interplay between PD and periodontal health.

Also, this year [41], a study explored the compositions of the oral microbiota, *P. gingivalis* copy number, and lysine gingival protein (Kgp) genotypes in PD patients with mild cognitive impairment (PD-MCI), PD patients with normal cognition (PD-NC), and a control group with similar periodontal status (PC). Although the alpha diversity indices showed no significant differences, the beta diversity indices, especially at the family and genus level, showed remarkable differences distinguishing PD-MCI from PD-NC and PC. *Prevotella*, *Lactobacillus*, *Megasphaera*, *Atopobium*, and *Howardella* were significantly elevated in the gingival crevicular fluid (GCF) of the PD-MCI group and negatively correlated with cognitive scores. The study also revealed significant differences in oral microbiota structure and Kgp genotypes between the groups, with a predominance of the Kgp II genotype in PD-MCI, which correlated with lower cognitive scores. These findings suggested a potential role for *P. gingivalis* in cognitive impairment in PD-MCI.

### 4.2. Animal Model Studies

Besides studies with human subjects, research employing animal models has generated important evidence indicating the involvement of the oral microbiota in PD (Table 2).

A 2020 study [42] investigated the involvement of oral *Porphyromonas gingivalis* (Pg)-induced inflammation in the pathophysiology of LRRK2-associated Parkinson’s disease using a PD mouse model. It was shown that oral Pg caused a mild inflammatory response in the gut, resulting in a significant loss of dopaminergic neurons and marked microglial activation in the SNpc. Furthermore, Pg ingestion triggered an immune response that involved IL-17A in the peripheral system and increased IL-17RA protein levels in dopaminergic neurons, indicating a possible connection between Pg, IL-17A, and dopaminergic neurodegeneration in LRRK2-associated PD. The negative impacts of oral Pg were accompanied by an elevation in LRRK2 expression, indicating the involvement of LRRK2 kinase in Pg-induced neuropathogenesis in LRRK2-associated PD. These results emphasized the interference of intestinal permeability caused by oral Pg, the rise in peripheral IL-17A levels in R1441G mice, and its potential contribution to neuronal death and neuroinflammation.

A 2023 study [43] demonstrated the exacerbating effects of oral pathogens on motor dysfunction and neurodegeneration in MPTP-induced PD mice in the context of periodontitis. Oral pathogens contributed to oral and intestinal dysbiosis, as well as brain and systemic immune activation, which exacerbated PD progression. The study identified the pivotal role of *V. parvula* and *S. mutans* in altering both oral and gut microbiota, potentially resulting in the infiltration of Th1 cells in peripheral and brain regions. This could result in increased microglial activation and contribute to neurodegeneration. Furthermore, the analysis of lipopolysaccharide-stimulated *Porphyromonas gingivalis* (LIP-SP) showed that it promoted the progression of PD in an environment that mimicked that of a periodontitis patient. LIP-SP exacerbated motor dysfunction, dopaminergic neuron loss, and microglial activation in their model. These findings bore implications for future research, shedding light on the role of oral pathogens in exacerbating PD and offering a potential strategy for disease prevention and treatment.

**Table 2:** This table summarizes the key studies investigating the oral microbiota in PD in human and animal model studies. The information includes the authors, year of publication, type of population studied, and the main findings of each study.

Authors	Year	Population	Key Findings/Main Conclusions
Pereira et al. [15]	2017	PD patients vs. Healthy controls	Significant differences in beta diversity and abundance of specific bacterial taxa between PD patients and controls. Increased abundance of *Prevotella*, *Prevotellaceae*, *Veillonella*, *Solobacterium*, *Veillonellaceae*, *Lactobacillaceae*, *Coriobacteriaceae*, *Moryella*, and *Erysipelotrichaceae* in PD patients. Decreased abundance of *Capnocytophaga*, *Rothia*, *Kingella*, *Leptotrichia*, *Actinomyces*, *Leptotrichiaceae*, *Haemophilus*, *Neisseria*, *Gemella*, *Corynebacterium*, and *Granulicatella* in PD patients. Males showed higher levels of potential oral pathogens than females, regardless of periodontal disease status. *Porphyromonas* Otu000029 and unclassified *Prevotellaceae* OTU positively correlated with age, while *Flavobacteriaceae* abundance negatively correlated with age.
Mihaila et al. [16]	2019	PD patients vs. Healthy controls	No differences in overall microbial diversity between PD patients and controls. Significant abundance changes in 50 taxa, including increased *Lactobacillus*, *Bifidobacterium*, *Candida species*, and decreased *Buchnera* and *Campylobacter* in PD patients. Metabolic shifts in amino acid and energy pathways. Strong correlations between microbiota and cognitive and motor measures in PD.
Fleury et al. [17]	2021	PD patients vs. Healthy controls	PD patients showed lower alpha diversity in both plaque and saliva samples. Salivary microbial profiles correlated with cognitive status, salivary flow rate, and increased risk for periodontal disease. Despite no significant differences in dental and periodontal parameters, distinct microbial compositions were observed in both saliva and plaque of PD patients compared to controls. PD patients exhibited higher abundance of specific bacteria such as Streptococcus mutans, *Kingella oralis*, and *Lactobacillaceae*, while others like *Treponema* KE332528 and *Lachnospiraceae* AM420052 were less abundant.
Jo et al. [22]	2022	PD patients vs. Healthy controls	PD patients have a higher abundance of oral *Lactobacillus* compared to healthy controls. Oral *Lactobacillus* showed the highest LDA score in PD patients, regardless of confounding factors. Increased oral *Lactobacillus* in PD patients is associated with higher levels of intestinal pathogens (*Citrobacter*, *Klebsiella*, *Enterobacter*). The specific influence of oral *Lactobacillus* on PD pathogenesis remains unclear.
Li et al. [33]	2022	PD patients vs. Healthy controls	Beta diversity analysis revealed distinct microbiota structures in the gut and oral cavity of PD patients. PD patients exhibited higher levels of *Firmicutes*, *Bacilli*, *Lactobacillales*, *Streptococcaceae*, *Streptococcus*, *Streptococcus mitis*, unidentified *Prevotellaceae*, and *Prevotellaceae* in their oral microbiota compared to controls. Controls showed higher abundance of *Neisseria*, *Neisseria subflava*, unidentified *Gammaproteobacteria*, *Neisseiaceae*, *Gammaproteobacteria*, and *Proteobacteria* compared to PD patients. Genera in the oral microbiota correlated positively with clinical features such as scores on the Hamilton Anxiety Scale (HAM-A) and Hamilton Depression Scale (HAM-D).
Kennedy et al. [34]	1994	PD patients who crave sweets vs. PD patients who do not crave sweets vs. Healthy controls	No correlation between oral microbiota and clinical assessments. Higher percentage of *Streptococcus mutans* in PD patients compared to controls. *Actinomyces*, *Streptococcus mutans*, and *Veillonella* were more frequent than *Lactobacillus*, *Bacteroides*, and *Fusobacteria*.
Gosney et al. [35]	2003	PD patients	16 PD patients had six types of Gram-negative bacteria: *Escherichia coli*, *Klebsiella* spp., *Kluyvera* spp., *Serratia* spp., *Proteus* spp., and *Enterobacter* spp. Higher prevalence of these bacteria in PD patients with oromuscular dysfunction vs. normal swallowing. No correlation between disease severity and the presence of these bacteria.
Rozas et al. [36]	2021	PD patients vs. Healthy controls	No significant differences alpha diversity between PD patients and controls. Significant beta diversity differences between hard and soft tissues in both groups. PD patients showed increased abundance of *Lactobacillus*, *Tannerella forsythia*, and *Prevotella intermedia*, alongside potential pathogenic species like *Streptococcus pneumoniae*, *Mycoplasma orale*, and *Streptococcus constellatus*. Dysphagia, drooling, and salivary pH significantly influenced soft tissue beta diversity and microbiota composition. Elevated *Streptococcus pneumoniae* levels were linked to PD patients with dysphagia compared to controls. PD severity and medication status impacted oral microbiota composition.
Zapala et al. [37]	2022	PD patients vs. Healthy controls	PD patients showed reduced levels of *Proteobacteria*, *Pastescibacteria*, and *Tenercutes*. Higher levels of *Prevotella*, *Streptococcus*, and *Lactobacillus* were found in PD patients. *Veillonella rogosae* was positively correlated with leafy greens consumption, and *Prevotella pallens* was negatively correlated with alcohol consumption.
Yay et al. [38]	2023	PD patients with periodontitis vs. Periodontitis patients vs. Healthy controls	Higher levels of specific bacteria (e.g., *Treponema socranskii*, *P. nodatum*, *P. micra*) were found in PD patients with periodontitis. PD patients with periodontitis showed increased levels of certain bacteria (e.g., *C. rectus*, *T. denticola*) in both deep and shallow pockets. Minimal differences in alpha and beta diversity between PD and PD with periodontitis groups. Periodontal disease could affect the subgingival microbiota in PD patients.
Arıkan et al. [39]	2023	PD patients with mild cognitive impairment vs. PD patients with full dementia vs. Healthy controls	Significant shifts in the salivary microbiome, protein translation, and defense mechanisms were observed. No significant differences in alpha diversity were apparent. Notable distinctions in beta diversity across cognitive impairment stages were present. There was a significant decrease in the *Neisseria* genus with advancing CI in PD. The findings highlighted the link between CI progression in PD and changes in salivary microbial communities and protein expression.
Yay et al. [40]	2024	PD Patients with periodontitis vs. PD patients without periodontitis vs. Healthy controls	There were significant beta diversity differences in saliva samples across groups. There were increased levels of *Mycoplasma faucium*, *Tannerella forsythia*, *Parvimonas micra*, and *Saccharibacteria* (TM7) in the Periodontitis group. There were higher abundance levels of Prevotella pallens, *Prevotella melaninogenica*, and *Neisseria* multispecies in the periodontitis + PD group. The study highlighted the impact of PD on the oral microbiome associated with periodontitis and the importance of demographic and clinical factors.
Li et al. [41]	2024	PD patients with mild cognitive impairment vs. PD patients with normal cognition vs. Periodontal status-matched control group vs. Healthy controls	No significant differences in alpha diversity among groups were found. Significant beta diversity differences at family and genus levels were found, distinguishing PD-MCI from PD-NC and PC. Elevated levels of *Prevotella*, *Lactobacillus*, *Megasphaera*, *Atopobium*, and *Howardella* in the PD-MCI group were negatively correlated with cognitive scores. Significant differences in the oral microbiome structures and Kgp genotypes were found. *P. gingivalis* played a potential role in cognitive impairment in PD-MCI.
Feng et al. [42]	2020	FVB/NJ and FVB/N-Tg (LRRK2*R1441G)135Cjli/J mice	Oral Pg induced mild gut inflammation, significant loss of dopaminergic neurons, and microglial activation in SNpc in a PD mouse model. Pg ingestion triggered an immune response involving IL-17A and increased IL-17RA in dopaminergic neurons. Elevated LRRK2 expression accompanied Pg-induced neurodegeneration. The findings highlighted the role of Pg, IL-17A, and LRRK2 in dopaminergic neurodegeneration and neuroinflammation in LRRK2-associated PD.
Bai et al. [43]	2023	C57BL/6 mice injected w/1-Methyl-4-phenyl-1,2,3,6-tetrahydropyridine (MPTP) to induce PD and ligature-induced periodontitis with the application of subgingival plaque (LIP-SP)	Oral pathogens exacerbated motor dysfunction and neurodegeneration in MPTP-induced PD mice with periodontitis. Oral pathogens caused oral and intestinal dysbiosis and brain and systemic immune activation, worsening PD progression. *V. parvula* and *S. mutans* played key roles in altering microbiota and promoting Th1 cell infiltration and microglial activation. Lipopolysaccharide-stimulated *Porphyromonas gingivalis* (LIP-SP) worsened motor dysfunction, neuron loss, and microglial activation.

## 5. Discussion

PD is a neurodegenerative condition that results from a complex interplay between genetic and environmental factors. Recent studies suggest that the oral and nasal microbiomes may play a role in the development and/or progression of PD. Their findings suggest that PD is generally associated with significant changes, particularly in the oral microbiota. Most of these changes are characterized by reduced alpha diversity and altered beta diversity. A deeper understanding of the mechanisms by which these microbiomes influence disease pathogenesis, along with further research into the bidirectional communication between them and their impact on PD, has the potential to reveal novel insights into disease pathophysiology and therapeutic avenues.

The nasal microbiota has received growing attention as a possible contributor to PD pathology. According to the “dual-hit hypothesis”, the nasal cavity may serve as a gateway for pathogen invasion or toxin exposure, resulting in neuroinflammation and disease progression [27]. A limited number of studies have suggested that the nasal microbiota of PD patients often shows decreased alpha diversity, which is potentially linked to the neurodegenerative PD processes. Additionally, research has identified aSyn immunoreactive inclusions in the nasal cavity of PD patients [24,44,45], suggesting a potential involvement of the nasal microbiome in initiating and spreading Lewy body pathology. Nevertheless, the evidence supporting the significant role of the nasal microbiota in the pathogenesis of PD remains scarce. Research into the nasal dysbiosis of PD produces inconsistent results. Some studies report no significant differences between PD patients and controls, while others have identified dysbiotic and pro-inflammatory PD nasal microbiota [15,29,30,31]. The connection between the composition of the nasal microbiome and PD remains inconclusive, primarily due to the scarcity of studies and participants. Further research is essential to elucidate the role of the nasal microbiome in PD’s pathophysiology and its correlation with significant clinical features like hyposmia and its response to deep brain stimulation.

The oral cavity is known to harbor a diverse microbial community that is critical for maintaining both oral and systemic health. Alterations in the oral microbiota have been implicated in several systemic diseases (e.g., cardiovascular disease and diabetes), including PD [46]. Unlike the findings on the nasal microbiota, research on the oral microbiota presents a more consistent picture. Several studies have shown that PD patients often have reduced alpha diversity in their oral microbiomes compared to healthy controls. This reduced diversity may be due to several factors, including altered immune responses, medication, and changes in diet or oral hygiene practices. These differences have the potential to impact the onset and progression of the disease. PD patients exhibit reduced microbial diversity in both saliva and plaque samples, accompanied by an increase in pro-inflammatory bacteria [17]. Several findings suggest a potential association between oral dysbiosis, inflammation, and the severity of PD motor symptoms. Additionally, the presence of local inflammation in the oral cavity of PD patients, as demonstrated by increased levels of inflammatory cytokines, highlights a plausible link between oral dysbiosis and the pathogenesis of PD [17].

Several studies have illuminated the dysregulation of the oral microbiota in PD [16,21,35]. These investigations have pinpointed correlations between specific microbial taxa and clinical manifestations of the disease. Moreover, periodontitis has emerged as a potential risk factor for PD [47], while the protective effect of dental scaling (a procedure to carefully target plaque and tartar accumulation on tooth surfaces, particularly below the gum line) against PD has been evidenced [48]. Also, non-surgical periodontal treatment seems to affect salivary aSyn levels and may be a confounding factor when using salivary aSyn as a biomarker in PD [49]. In addition, a positive association between tooth loss and PD onset has been established [50], while gingipain R1 (RgpA), produced by *P. gingivalis*, has been detected in blood clots from PD patients [51], suggesting a connection between periodontal disease and PD. Consequently, periodontitis, which can initiate systemic inflammation, a hallmark of PD, represents a promising avenue for mitigating neuroinflammation and preventing neurodegenerative diseases, such as PD and other alpha-synucleinopathies [21,52,53]. Furthermore, recent animal studies [42,43] have highlighted the role of oral pathogens in the pathophysiology of PD. These studies demonstrate that oral pathogens have the potential to induce neuroinflammation and dopaminergic neurodegeneration.

The oral and nasal cavities contain different microbial communities that might communicate with each other. Studies have shown that the microbial compositions in both oral and nasal cavities of PD patients differ from those of healthy individuals. Oral and nasal microbiota may have significant impacts on the onset and progression of PD and may also affect disease pathogenesis. The mechanisms behind these interactions are not yet clear, but they may involve direct pathogen invasion or microbial metabolite production, which can trigger neuroinflammation and aSyn aggregation. Additionally, the potential migration of oral pathogens to the gut and subsequent alterations in the intestinal environment is emphasized in a recent study [22], underscoring the complex interplay between the oral and gut microbiomes in PD pathogenesis and emphasizing conditions that are linked to damage to the enteric nervous system. Oral dysbiosis can potentially impact the gut microbiome [33], leading to gastrointestinal dysfunction, a common non-motor symptom of PD. Remarkably, 45% of participants in the Human Microbiome Project exhibit a correlation between their oral and fecal bacteria [16]. However, it is important to note that although certain bacterial taxa have shown significant differences across several studies, the reported alterations are not entirely consistent overall. Variability in the results may be due to differences in the inclusion criteria of PD patients, disease severity, sequencing methodologies, geographic variations, and the handling of confounding factors.

The changes observed in the oral and nasal microbiotas in PD have significant clinical relevance, presenting potential diagnostic markers and potential opportunities for therapeutic interventions. The use of microbiome-based biomarkers has the potential to facilitate the early detection of PD, particularly during the pre-motor phase. Therapeutic strategies aimed at modulating the microbiome offer promising avenues for treating PD, including interventions such as probiotics, prebiotics, dietary modifications, or personalized microbiome-based therapies to mitigate disease progression [54]. However, translating microbiome-based interventions for PD into clinical practice presents challenges in terms of specificity, efficacy, and long-term impact on disease progression.

Future research in PD should prioritize the exploration of the complex interplay between the microbiome and PD. Longitudinal studies tracking temporal changes in the composition and function of the oral, nasal, and gut microbiota of PD patients hold great promise for elucidating PD progression and its correlation with microbiome dynamics. The use of animal models to replicate the microbiome changes observed in PD patients is also an important avenue. These models enable researchers to investigate the mechanistic underpinnings of microbiome-induced effects on PD progression. Such studies provide critical insights into the causal relationships between microbial dysbiosis and disease manifestations. Advanced microbiome analysis techniques, such as metagenomics, metatranscriptomics, and metabolomics, provide unparalleled opportunities to enhance our comprehension of microbial interactions in PD pathogenesis. These advanced methods enable researchers to conduct comprehensive analyses of microbial communities, their gene expressions, and metabolite profiles within the host environment. This facilitates the exploration of complex microbial interactions and their impacts on host physiology, aiming to uncover new therapeutic targets and diagnostic biomarkers for PD.

Initial findings indicate a potential connection between microbiota dysbiosis and PD, but further research is necessary to thoroughly understand this relationship and its clinical significance.

## 6. Conclusions

The study of the nasal and oral microbiome may offer interesting insights into the etiology of PD.

Research into the role of nasal microbiota in PD underscores its complexity, yet the scarcity of studies limits conclusive insights. The “dual-hit hypothesis” underscores the necessity for ongoing investigation. The conflicting findings regarding the association between nasal microbiota and PD raise questions about the reliability of nasal microbiota as a PD biomarker.

Oral microbiota reveals significant differences in PD patients, suggesting a possible link to disease onset and progression. These findings, supported by evidence from animal models, indicate a potential role for oral pathogens in neuroinflammation and motor symptoms.

**Figure 2:**
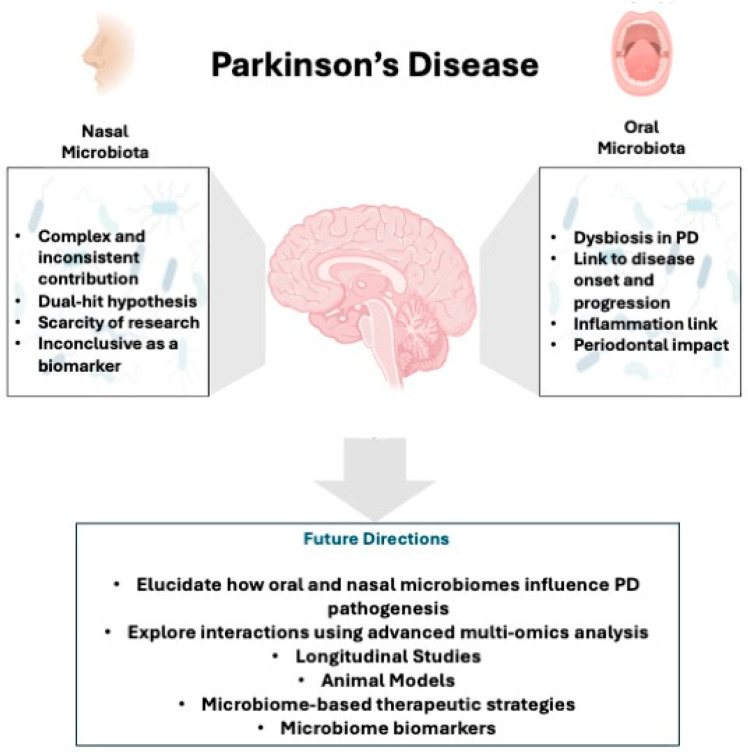
The summary figure provides an overview of key findings from the reviewed literature and future directions in the study of nasal and oral microbiota in PD.

Understanding the complex interplay between nasal and oral microbiota and PD pathophysiology is essential for the development of treatment strategies. Further research is needed to unravel the underlying mechanisms and identify potential therapeutic targets that hold promise for improving outcomes for individuals with PD [55] (Figure 2).
